# The role of astrocytes in epileptic disorders

**DOI:** 10.14814/phy2.15239

**Published:** 2022-03-28

**Authors:** Parichehr Hayatdavoudi, Mahmoud Hosseini, Vahid Hajali, Azar Hosseini, Arezoo Rajabian

**Affiliations:** ^1^ 37552 Applied Biomedical Research Center Mashhad University of Medical Sciences Mashhad Iran; ^2^ 37552 Department of Physiology Faculty of Medicine Mashhad University of Medical Sciences Mashhad Iran; ^3^ 37552 Division of Neurocognitive Sciences, Psychiatry and Behavioral Sciences Research Center Mashhad University of Medical Sciences Mashhad Iran; ^4^ 37552 Department of Neuroscience Faculty of Medicine Mashhad University of Medical Sciences Mashhad Iran; ^5^ 37552 Pharmacological Research Center of Medicinal Plants Mashhad University of Medical Sciences Mashhad Iran; ^6^ 37552 Department of Pharmacology Faculty of Medicine Mashhad University of Medical Sciences Mashhad Iran; ^7^ 37552 Department of Internal Medicine Faculty of Medicine Mashhad University of Medical Sciences Mashhad Iran

**Keywords:** antiepileptic, astrocyte, epilepsy, glia, seizure

## Abstract

Epilepsy affects about 1% of the population and approximately 30% of epileptic patients are resistant to current antiepileptic drugs. As a hallmark in epileptic tissue, many of the epileptic patients show changes in glia morphology and function. There are characteristic changes in different types of glia in different epilepsy models. Some of these changes such as astrogliosis are enough to provoke epileptic seizures. Astrogliosis is well known in mesial temporal lobe epilepsy (MTLE), the most common form of refractory epilepsy. A better understanding of astrocytes alterations could lead to novel and efficient pharmacological approaches for epilepsy. In this review, we present the alterations of astrocyte morphology and function and present some instances of targeting astrocytes in seizure and epilepsy.

## Graphical abstract

As a hallmark in epileptic tissue, many of the epileptic patients show changes in glia morphology and function. A better understanding of astrocytes alterations helps finding an efficient pharmacological approaches for epilepsy. Considering the alterations of astrocyte morphology and function, astrocytes may be considered as targets in seizure and epilepsy.

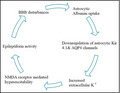

## 1 INTRODUCTION

Repeated unprovoked epileptic seizures are defined as epilepsy (Thijs et al., 2019). Epilepsy affects about 46 million people in the world (Beghi, 2020). Epileptogenesis occurs when the neuronal network shifts to make recurrent seizures after an initial insult or makes more vigorous frequent seizures in chronic epilepsy (Engel & Pitkänen, 2020). Dysregulation of water and ion channel expression, variations in the secretion of neuroactive molecules, and increased activation of inflammatory pathways, as well as reactive gliosis, are characteristic features in epilepsy (Nickels & Noe, 2021; Rajabian et al., 2022). Considerable alteration in shape and function of glial cells occurs in various kinds of epilepsy. The central nervous system (CNS) is composed of different kinds of glial cells including astrocytes, oligodendrocytes, ependymal cells, microglia, and Bergmann glia (Foresti et al., 2011; Patel et al., 2019). Glial cells may direct a feeding mechanism, signal transduction from blood to neurons, removal of synaptic glutamate, neuronal path finding, and the sequestration and redistribution of K^+^ ion (Foresti et al., 2011; Trosclair et al., 2021). The K^+^ long‐range spatial buffering conducted by glia is a parallel synchronizing and/or spreading mechanism during paroxysmal oscillations (Chung et al., 2013). Glial cells deliver neuroactive molecules and adjust synaptic transmission through modifications of ion channels, gap junctions, receptors, and transporters (Binder & Carson, 2013).

There is a bidirectional flow of information between neurons and glial cells in the CNS (Héja et al., 2012; Matejuk & Ransohoff, 2020). A variety of processes called neurotransmitter cycles happen at the neuroglial connections between the glutamatergic and gamma‐aminobutyric acid (GABA)‐ergic synapses. For instance, the cytoplasm of astrocytes contains brain glutamine synthetase (GS) which is particularly enriched in astrocyte processes in specific parts of the neuropil. GS is the only enzyme in the mammalian brain which converts glutamate and ammonia to glutamine (Alemanno, 2020; Mazaud et al., 2019). The intracellular localization of GS in astrocytes is altered in mesial temporal lobe epilepsy (MTLE) (Eid et al., 2013). Furthermore, astrocytes end‐feet around vessels contribute to making the blood–brain barrier (BBB) which prevents the entry of most blood constituents to the brain. It has been argued that direct contact between astrocytes and endothelial cells or humoral factors released from astrocytes is required to induce BBB properties (Heithoff et al., 2021; Kadry et al., 2020). It has also been assumed that permeability alteration of BBB is involved in epileptogenesis (Dadas & Janigro, 2019).

### 1.1 Astrocytes in epilepsy

There is some evidence that astrocytes have a role in seizure and epilepsy. It has been suggested that epilepsy has an astrocytic origin because direct stimulation of astrocytes causes prolonged neuronal depolarization and epileptiform discharges (Chan et al., 2019; Fellin & Haydon, 2005). Reactive astrocytes in epileptic tissue either promote or inhibit seizure development through different specific mechanisms (Chan et al., 2019; Wetherington et al., 2008). For example, downregulation of inward rectifier K^⁺^ channels (Kir4.1 channel) characterizes transformed astrocytes in the epileptogenic tissue (Chu‐Shore et al., 2010; Kinboshi et al., 2020) and aquaporin (AQP4)‐null mice show reduced [Ki]_o_ buffering and prolonged duration of induced seizures (Binder et al., 2006; Strohschein et al., 2011). A better understanding of astrocytes alterations could lead to novel and efficient pharmacological approaches for epilepsy. In this review, we underline the alterations of astrocytes and summarize some instances of targeting astrocytes in seizure and epilepsy.

## 2 ASTROGLIOSIS AND ASTROGLIAL DEATH IN EPILEPSY

Astrogliosis refers to the morphological, biochemical, and functional changes of astrocytes that occur in response to brain insults or injuries. The changes include hypertrophy and up‐regulation of the intermediate filament glial fibrillary acidic protein (GFAP), reversible alterations in gene expression, and pronounced cell proliferation with compact scar formation and permanent tissue rearrangement (Robel & Sontheimer, 2016; Sofroniew, 2014). Astrogliosis is well known in MTLE, the most common form of refractory epilepsy (Robel & Sontheimer, 2016). One of the typical features of temporal lobe epilepsy and other epilepsy syndromes is hippocampal sclerosis (Lee et al., 2021). In the sclerotic hippocampus, for instance, Na^⁺^ channels are augmented while Kir4.1 channels are reduced; thus, action potential generation is adjusted. Furthermore, the glutamine synthetase is decreased and glutamate dehydrogenase is increased in the sclerotic hippocampal tissue. The expression of many inflammatory and immune‐related molecules is also upregulated in astrocytes (Aoki et al., 2019; Chan et al., 2019). It also seems that the chemical signaling in epileptic tissue is augmented, the association of water and K^⁺^ equilibrium is disturbed and the microenvironment at the border between astrocytes and microvasculature is deteriorated (Wetherington et al., 2008).

The morphological, biochemical, and functional modifications which occur in astrogliosis may make astrocytes resistant to different harmful stimuli. Astroglial death has also been reported following status epilepsy (SE) and kainic acid‐induced epilepsy in CA1 (Ko et al., 2016; Revuelta et al., 2005). Astroglial apoptosis (Hyun et al., 2017) and autophagic astroglial death (clasmatodendrosis) have also been reported after SE in the molecular layer of the dentate gyrus and the stratum radiatum in the CA1 (Ryu et al., 2011). The regional‐specific astroglial death is independent of hemodynamics. Evidence indicates that the differential mitochondrial dynamics in astrocytes play a key role in the regional‐specific astroglial death. The mitochondrial dynamics is highly correlated with dynamin‐related protein 1(DRP‐1) which is a mitochondrial fission protein (Ko et al., 2016). Inhibition of DRP‐1 has exerted protective effects on hippocampal neurons in pilocarpine‐induced seizures in rats possibly through reducing the cytochrome c (Cyt C) release, apoptosis‐inducing factor (AIF) translocation, and prevention of mitochondrial‐dependent apoptosis pathway (Xie et al., 2013).

## 3 ASTROCYTES AND GLUTAMATE/GABA TRANSPORTERS IN EPILEPSY

Neuron–astrocyte interactions are important in the excitatory/inhibitory balance. Interruption of this balance may play a role in the abnormal neuronal activity in seizures (Verdugo et al., 2019). Glutamate released from astrocytes not only enhances neuronal irritability by a feed‐forward mechanism during seizure‐like events (SLE) but also affects the Hebbian plasticity at single synapses (Perea & Araque, 2007) and produces coordinated activity in neuronal pools (Fellin & Haydon, 2005; Mederos et al., 2018). Following BBB dysfunction, activated astrocytes show reduced levels of mRNA encoding for the astrocytic glutamate transporters of the solute carrier family 1 subfamily A (SLC1A) members, SLC1A2 and SLC1A3. The enzymes such as glutaminase and glutamine synthetase are also down‐regulated in astrocytes (Heinemann et al., 2012; Swissa et al., 2019). Reduction of the glutamate uptake in transformed astrocytes may also interrupt the production of glutathione. Astrocytes use glutamate to uptake cystine for synthesizing glutamylcysteine, which is released from astrocytes for the synthesis of glutathione in neurons. Down‐regulation of neuronal and glial glutathione would lessen the defense mechanisms against free radicals and may result in increased damage. Furthermore, glutamine decrement may impede the detoxification of ammonium. Ammonium disturbs Cl2 transporters and may result in the reduction of the GABA‐mediated synaptic inhibition (Heinemann et al., 2012; Swissa et al., 2019).

On the other hand, it has been shown that glutamate uptake leads to GABA release from astrocytes which directly affects the irritability action of hippocampal pyramidal neurons. Astrocytic GABA release is mediated by the reverse action of glial GABA transporter (GAT) subtypes, GAT‐2 or GAT‐3. The activity of the glutamate transporter triggers the reversal of GABA transporters through increasing astrocytic Na^⁺^ concentration. Then, GABA causes tonic inhibition in a network activity‐dependent way. This is an example of an in situ negative feedback mechanism by which astrocytes convert the glutamatergic excitation to GABA‐ergic inhibition for modifying the excitability of neurons (Héja et al., 2012).

Glutamate and GABA uptake by astrocytes is an example of active hyperemia that induces a local increase in cerebral blood flow (CBF) through a variety of mechanisms. Interestingly, astrocytes exert a slow indirect role in these mechanisms whereas the neurons play a fast direct effect (Attwell et al., 2010; Banks et al., 2018; Marina et al., 2020). The association of calcium dynamics in CBF regulation could explain the major involvement of astrocytes (80%) rather than neurons in CBF. Indeed, the evidence showed that the vast majority of astrocytes responded with a calcium elevation to ictal but not interictal discharges (Gómez‐Gonzalo et al., 2010). Using a computational method, it was found that when the interictal discharge was sufficiently important, astrocytes contribution was already present (Blanchard et al., 2016).

## 4 ASTROCYTES, GAP JUNCTIONS, CONNEXINS, AND PANNEXINS IN EPILEPSY

Gap junctions (GJ) have been found to play an important role in neuronal synchronization and seizure induction (Fonseca et al., 2002; Li et al., 2019; Onodera et al., 2021). Knocking out (dKO) of the glial connexin (Cx) 30 and 43 has revealed that GJ communication between astrocytes is required for glucose or lactate transport to astrocytes which is essential to maintain excitatory synaptic transmission and epileptiform activity (Li et al., 2019; Rouach et al., 2008; Wallraff, 2006). GJ communication is also involved in propagating apoptotic signals (Y. Wang et al., 2012), a process that is significant in severe seizure activity (Engel & Henshall, 2009). Recently, using dKO mice it was revealed that animals lacking oligodendrocytic Cx32 and astrocytic Cx43 displayed seizures, motor impairment, and early mortality (Magnotti et al., 2011). Investigations of GJ expression in epileptic brains in humans either reported no change (Elisevich et al., 1997) or elevated levels of glial Cx mRNA and protein (Collignon et al., 2006; Naus et al., 1991). The role of pannexins has not been studied as much as Cx. Studies suggest that blocking Panx1 channels reduces excitability and can be anticonvulsant. They may also exert compensatory, overlapping, or exclusive physiological roles compared to those of Cx in seizure models (Aquilino et al., 2019).

## 5 ASTROCYTES AND CYTOKINES IN EPILEPSY

In contrast to the well‐known role of microglia as antigen‐presenting cells (APCs), the role of astrocytes in antigen presentation is still unclear (Aronica et al., 2012). Studies suggested that while microglia may activate both Th1 and Th2 cells (T helper 1 and 2 cells, respectively), astrocytes mainly stimulate Th2 responses, providing homeostatic mechanisms which may limit brain inflammation (F. Aloisi et al., 2000; Francesca Aloisi et al., 1998). Astrocytes have been shown to initiate, regulate, and amplify the immune‐mediated mechanisms involved in different CNS diseases including epilepsy (Kwon & Koh, 2020; Seifert et al., 2010). They are also the target of inflammatory molecules which may aggravate astrogliosis and intensify the pro‐epileptogenic inflammatory signaling through the activation of specific receptors and related signaling pathways (Giovannoni & Quintana, 2020; Kwon & Koh, 2020). Based on in vitro studies, astrocytes (particularly reactive astrocytes) can generate cytokines such as interleukin (IL)‐1β, IL‐6, tumor necrosis factor (TNF)‐α, transforming growth factor‐beta (TGF)‐β, and chemokines such as monocyte chemoattractant protein‐1 (MCP‐1) and chemokine C‐ motif ligand 2 (CCL2), which are highly expressed in both the experimental and human epileptogenic brain tissue (Aronica et al., 2012; Giovannoni & Quintana, 2020). In an inflammatory epileptic encephalopathy of childhood, that is, Rasmussen's encephalitis (RE), expression of major histocompatibility complex (MHC) class I molecules have been reported to increase in astrocytes (Bauer et al., 2007). Therefore, an MHC class I‐restricted T‐cell response has been proposed as a possible mechanism for the astrocytic breakdown in RE (Bauer et al., 2007).

Reactive astrocytes also contain complement components and express complement‐regulatory proteins as well as complement receptors (Farina et al., 2007; Giovannoni & Quintana, 2020). Cytokine production is adjusted by complement system products such as C3 and cytokines such as IL‐1β may induce complement factor expression in human astrocytes (Bonifati & Kishore, 2007; Morotti et al., 2018). Meanwhile, astrocytes induce inhibitory factors such as complement factor H (CFH) which can modify the inflammatory pathway (Giovannoni & Quintana, 2020; Griffiths et al., 2009). An extensive and complex cross‐talk between complement and Toll‐like receptors (TLRs) has been proposed (Hajishengallis & Lambris, 2010; Kumar, 2019). Upregulation of IL‐1R1 or TLRs in reactive astrocytes in the human brain has been reported in epilepsy (Aronica et al., 2012). It has been demonstrated that the TLR4 and its endogenous ligand, high mobility group box‐1 (HMGB1), are overexpressed in reactive astrocytes in human temporal lobe epilepsy (TLE) (Kan et al., 2019; Maroso et al., 2010). Following release from neurons, HMGB reacts with TLR4 to develop seizures which in turn induces an additional wave of HMGB1 release from activated astrocytes and microglia. Consequently, it leads to a positive feedback cycle of seizures and inflammation which can be the core mechanism of recurrent seizures (Vezzani et al., 2011).

## 6 ASTROCYTES AND PURINERGIC RECEPTORS IN EPILEPSY

Purinergic receptors play a critical role in neuron‐glia communication and neuroinflammation (Agostinho et al., 2020; Kovács et al., 2015). There is a general agreement that extracellular and synaptic adenosine (Ado) levels are mainly regulated by astrocytes. Ado and non‐Ado nucleosides may be transported through neuronal and glial cell membranes by two types of nucleoside transporters, the equilibrative nucleoside transporter family (ENT) and the sodium‐dependent concentrative nucleoside transporter family (CNT) (Young et al., 2013). The astrocytic cycle that maintains the extracellular Ado levels consists of the release of ATP that is broken down to Ado, direct release and uptake of Ado through ENTs, and conversion of intracellular Ado to Ado phosphates. Adenosine receptors are coupled with “inhibitory” G‐proteins (Gi) or “stimulatory” G‐proteins (Gs) such as A1/A3 and A2A/A2B receptors, respectively. The G‐protein‐coupled A1, A2A, A2B, and A3 receptors express on both neuronal and glial cells (Dias et al., 2013; Fredholm, 2012; Jennings et al., 2001; Kovács et al., 2011; Sperlágh & Sylvester Vizi, 2011; Zarrinmayeh & Territo, 2020). In animal models of human absence epilepsy in Wistar albino Glaxo/Rijswijk (WAG/Rij) (D'Alimonte et al., 2009) and genetic absence epilepsy rat from Strasbourg (GAERS) rats (Ekonomou et al., 1998), kainic acid‐induced epilepsy (Ekonomou et al., 2000), as well as in the epileptic temporal cortex in human (Glass et al., 1996), distribution of A2A and/or A1 receptor density is altered. Thus, Ado receptors play a role in epileptic activity (Kovács et al., 2015). The ENTs on astrocytes efficiently convert Ado to adenosine monophosphate (AMP) by adenosine kinase (ADK); thereby, astrocytes remove Ado from the extracellular space and stop adenosinergic signaling (Fredholm, 2012). ADK is predominantly localized to astrocytes and phosphorylation of ATP to AMP by ADK plays a major role in the Ado metabolism (Boison et al., 2010). The expression of ADK can be adjusted by inflammatory molecules, such as IL‐1β, providing potential modulatory crosstalk between the astrocyte‐based adenosine cycle and inflammation (Aronica & Crino, 2011).

## 7 ASTROCYTIC PH DYNAMICS IN EPILEPSY

Previously, neuronal acidification had been established during seizures (Raimondo et al., 2015, 2016). Recently, using genetically encoded pH sensors, it has been demonstrated that astrocytes are alkalinized during seizures. Furthermore, astrocytes have shown faster pH change than neurons. The alkalinization is correlated with changes in membrane potential and generated by an electrogenic Na^⁺^/HCO3ˉ co‐transporter. Moreover, the astrocytic pH alterations are more closely associated with network activity than neuronal pH changes (Raimondo et al., 2016).

## 8 ASTROCYTE DYSFUNCTION IN TUBEROUS SCLEROSIS COMPLEX

Tuberous sclerosis complex (TSC) is a multisystem genetic disorder due to autosomal dominant mutations of either the TSC1 or TSC2 genes which are among the most common genetic causes of epilepsy. Patients with TSC demonstrate epilepsy in 80–90% of cases. They demonstrate multiple seizure types which are often resistant to antiepileptic drugs (AEDs) (Gupta et al., 2020). The disease usually occurs in the first year of life (Curatolo et al., 2018). The number of astrocytes is increased in tubers compared to the control and also neighboring brain tissue. There are morphological and biological differences in the subpopulation of astrocytes in tubers (Sosunov et al., 2008). Astrogliosis in tubers is composed of a combination of gliotic astrocytes similar to what is seen in hippocampal sclerosis and also reactive astrocytes which are vimentin immunoreactive and show the mammalian target of rapamycin (mTOR) activity (Wong, 2019). Proteins encoded by TSC1 and TSC2 function as negative regulators of the mTOR signaling pathway. Therefore, loss of function mutations of either TSC1 or TSC2 is followed by constitutive mTOR activation (Wong & Crino, 2012). The phosphorylated S6 protein, a downstream mTOR substrate, has been observed in dysplastic astrocytes which confirms that astrocytosis could reflect direct effects of mTOR pathway activation (Talos et al., 2008). An increase in vascular endothelial growth factor A (VEGFA) expression has been reported within cortical tubers of people with TSC (Parker et al., 2011). Expression reduction of glutamate transporters, glutamine synthetase, and the Kir 4.1 has been also observed in gliotic astrocytes in tubers. Abnormalities in the astrocytic regulation of glutamate and potassium have also been recognized in animal models of TSC and human tuber specimens resected during epilepsy surgery (Talos et al., 2008; Wong & Crino, 2012).

## 9 BLOOD–BRAIN BARRIER DISRUPTION AND EPILEPSY

Following the BBB illness, the neuronal network restructures itself because of the renovation or activation of glia. In the case of the experimental disintegration of the BBB of the rat neocortex, the delayed development of paroxysmal hypersynchronous activity which is indicative of epileptogenesis is recorded ex vivo and in vivo. For instance, direct brain exposure to serum albumin leads to albumin uptake into astrocytes via transforming growth factor‐beta receptors (TGF‐β Rs). Albumin uptake is followed by down‐regulation of Kir4.1 and aquaporin 4 channels (AQP4) in astrocytes, resulting in reduced buffering of extracellular potassium (David et al., 2009; Heinemann et al., 2012). This, in turn, leads to activity‐dependent increased extracellular potassium, leading to facilitated *N*‐methyl‐d‐aspartate (NMDA)‐receptor‐mediated neuronal hyperexcitability and eventually epileptiform activity (Ivens et al., 2007; Seiffert et al., 2004).

**FIGURE 1:**
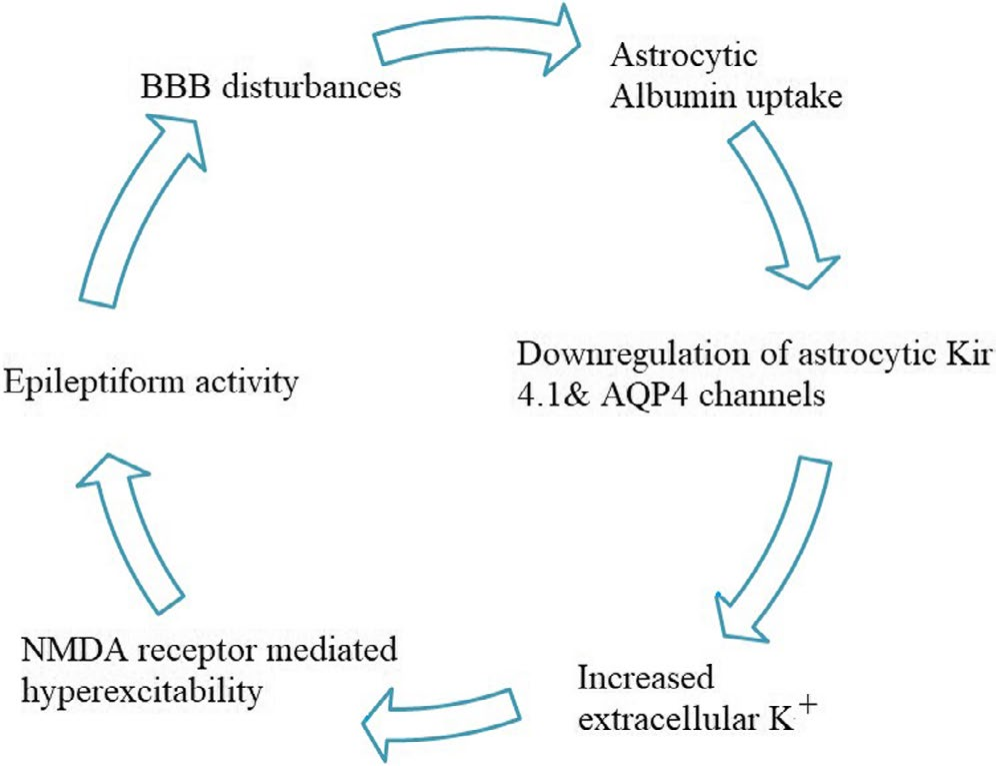
Schematic representation of the relationship between BBB disturbance and epileptiform activity

**FIGURE 2:**
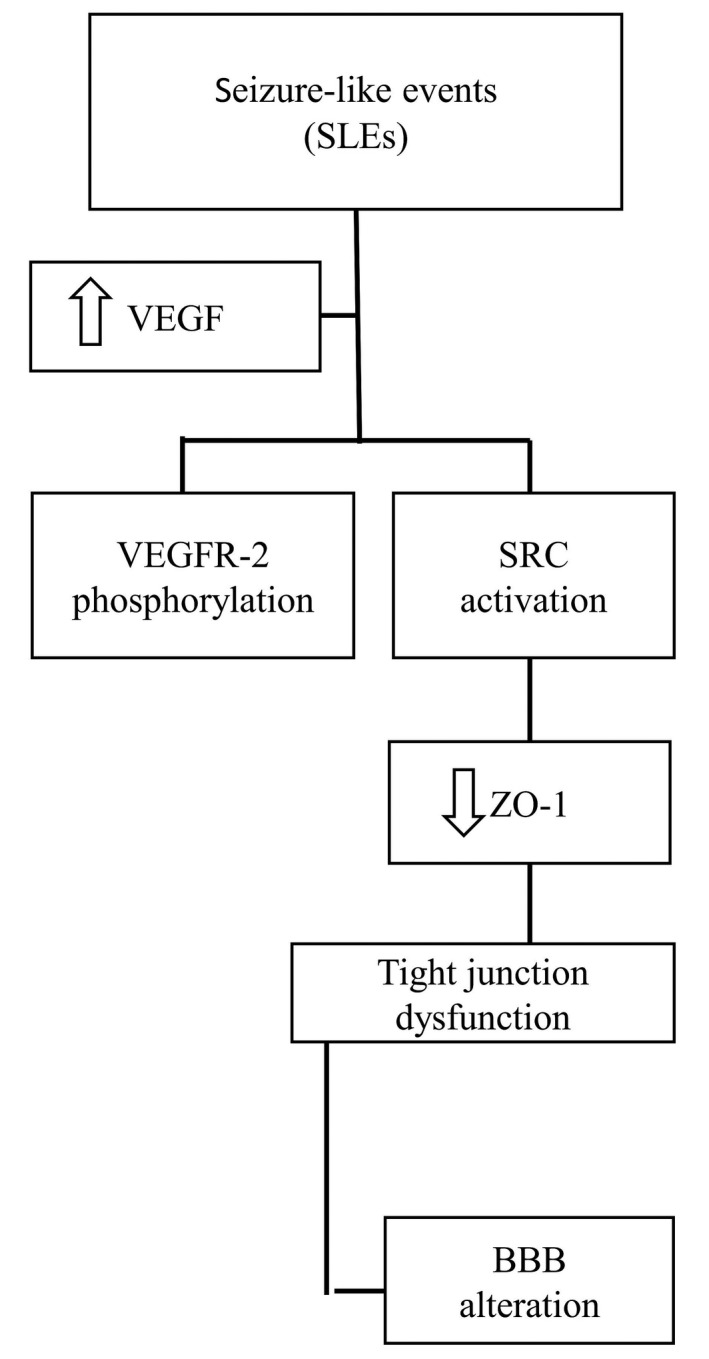
Cascade of events leading to blood brain barrier disruption after seizure like events. VEGF, vascular endothelial growth factor; VEGFR‐2, vascular endothelial growth factor receptor 2; SRC, xxx; ZO‐1, zonula occludense 1; BBB, blood brain barrier

The vascular lesion has also been proposed in the pathogenesis of post‐traumatic epilepsy (PTE) in humans (Sakai et al., 2018; Tomkins et al., 2011). It has been reported that the VEGFA is up‐regulated in reactive astrocytes in human epileptogenic tissue (B. Bauer et al., 2008; Morin‐Brureau et al., 2011; Rigau et al., 2007). The integrity of the BBB changes by VEGFA‐signaling pathways (Schoch et al., 2002; Thanabalasundaram et al., 2010). Following hypoxia or inflammation, VEGFA is increased by induction of transcription factors including hypoxia‐inducible factor‐1 (HIIF‐1), activation protein‐1 (AP‐1), specificity protein 1 (SP‐1), signal transducer, and activator of transcription factor‐3 (STAT3). Seizures can also induce all of these transcription factors in addition to VEGFR‐2 receptor activation (Morin‐Brureau et al., 2011). The endothelial cells and neurons express VEGFR‐2. VEGFA‐VEGFAR‐2 signaling leads to the variation of tight junctions and vascular remodeling via Src and PKC downstream pathways (Figures 1 and 2) (Morin‐Brureau et al., 2011).

## 10 ASTROCYTES AND INFLAMMATION AND IMMUNITY IN EPILEPSY

The beneficial effects of steroid‐containing and other anti‐inflammatory drugs in the treatment of epilepsy led to the hypothesis that inflammation plays a crucial role in epilepsy (Vezzani et al., 2011). Chronic brain inflammation was initially noted in RE, a kind of childhood epilepsy (Vezzani et al., 2011). Inflammation can be not only a cause but also a consequence of epilepsy (Vezzani et al., 2011). Seizure predisposition has shown a dramatic reduction in mice with overexpressed IL‐1Ra, an endogenous antagonist of IL‐1B, in astrocytes (Teresa Ravizza et al., 2006). Autoimmune disorders also led to seizure and epilepsy, for example, in RE, and in severe intractable seizures, glutamate receptor 3 antibodies have been seen (Mantegazza et al., 2002). There is astrocytic apoptosis and loss in cortical and white matter areas in RE and astrocytes express MHC class 1, moreover, granzyme‐B+ lymphocytes are close to astrocytes bordering astrocyte‐deficient lesions. Granzyme‐B+ granules have polarization facing the astrocytic membrane (J. Bauer et al., 2007).

Elevation of inflammatory cytokines production such as IL‐1β, IL‐6, and TNF‐α were reported in patients and animal models of TLE (Vezzani et al., 2011, 2013), in the hippocampus 1 day after SE induction by electric stimulation (De Simoni et al., 2000) and about 3 hours after developmental febrile SE (Patterson et al., 2015). Neurons, microglia, astrocytes, and endothelial cells produce inflammatory cytokines (Benson et al., 2015). Furthermore, astrocytes may play a role in the modulation of cytokine release from microglia (Hiragi et al., 2018). Following administration of TLR4 antagonist, acute seizures were reduced in kainic acid‐induced epilepsy in mice. It has been suggested that astrocytes express TLR4 following kainic acid‐induced seizures (Maroso et al., 2010).

## 11 ASTROCYTES IN FEBRILE SEIZURES

When a previously healthy child sustains severe refractory SE after a brief recovery from a short febrile disease, and infectious encephalitis is ruled out, then the condition can be suspected as febrile infection‐related epilepsy syndrome (FIRES) (van Baalen et al., 2017). FIRES is a sporadic condition. SE appear at the days following fever initiation in FIRES which is contrary to febrile SE. About 5% of SE cases are categorized as FIRES and in adults, these are called malignant or new‐onset SE (Vezzani et al., 2015).

It has been suggested that neuroinflammation plays a role in FIRES‐associated epileptogenesis in animal models of infection (Galic et al., 2012; Riazi et al., 2010), as well as febrile and afebrile SE later developing epilepsy (Dubé et al., 2010). Neuroinflammation reduces seizure threshold rather than triggering seizures, thus FIRES shows a delayed onset (van Baalen et al., 2017).

It has been indicated that astrocytes initiate, adjust and enhance immune‐mediated responses in CNS human diseases such as epilepsy (Farina et al., 2007; Seifert et al., 2010). It has been reported that astrocytes mediate mainly Th2 reactions, triggering homeostatic mechanisms to reduce brain inflammation (Aloisi et al., 2000).

## 12 ASTROCYTES AND HIPPOCAMPAL SCLEROSIS

One of the characteristic features of refractory temporal lobe epilepsy is hippocampal sclerosis (HS) (Sendrowski & Sobaniec, 2013). There is pyramidal cell loss in CA1, CA3 and around end‐folium in classical HS, however, CA2 cells are maintained. In certain kinds of HS, pyramidal cell loss occurs in all hippocampal fields (total hippocampal sclerosis), or only around end‐folium (end‐folium hippocampal sclerosis) (Thom, 2004). Furthermore, degeneration of neurons, gliosis, sprouting of mossy fibers, and dispersion of dentate gyrus granule cells are seen in HS (MD et al., 2002). It has been reported that astrocytes have a specific structure and function in HS (D. K. Binder & Steinhäuser, 2006). It has been reported that the activity of excitatory sodium currents is enhanced through the cell membrane of hippocampal astrocytes in patients with HS (Bordey & Spencer, 2004). Increased expression of genes encoding for proteins leading to glutamate release was noted on the surface of astrocytes in hippocampal samples of patients with HS (T.‐S. Lee et al., 2007).

## 13 TARGETING ASTROCYTES FOR THERAPEUTIC APPROACHES IN EPILEPSY

Because of the bidirectional flow of information between neurons and glial cells and glial–glial or glial microenvironmental compartments, there are various prospective strategies for developing/ testing new anti‐epileptic drugs. Of note, glutamate plays a pivotal role in the initiation and propagation of seizures. It has been suggested that increased glial glutamate transporter EAAT2 which enhances glutamate uptake, is a potential therapeutic approach for treating epilepsy. Kong et al. (2012) reported: (1) mortality rates decreased in EAAT2 transgenic mice after pilocarpine SE, (2) increased EAAT2 attenuated hippocampal neuronal loss after SE, (3) increased EAAT2 inhibited neurogenesis and mossy fiber sprouting after SE, and (4) increased EAAT2 reduced spontaneous recurrent seizures after SE (Kong et al., 2012).

Furthermore, astrocytes control the activity of Ado receptors that are expressed in neurons, astrocytes, microglia, and oligodendrocytes (Kovács et al., 2015). Adenosine and its analogs, together with non‐adenosine (non‐Ado) nucleosides (e.g., Guanosine (Guo), Inosine and Uridine) have shown anti‐seizure activity. Adenosine kinase inhibitors, Ado uptake inhibitors, and Ado‐releasing implants have also shown beneficial effects on epileptic seizures (Kovács, Kékesi, Juhász, Barna, et al., 2014; Kovács et al., 2015; Kovács, Kékesi, Juhász & Dobolyi, 2014). The Ketogenic diet, a therapeutic regimen in refractory epilepsy, reduces plasma glucose levels and also suppresses seizure through A1 receptor activation and reduction of ADK expression. The pharmacological blockade of ADK has also exerted a powerful antiepileptic effect in several models of epilepsy (Kovács et al., 2015; Wang & Li, 2020). Additionally, activation of Guo receptors may reduce extracellular glutamate level and glutamate‐induced excitability by triggering glial glutamate uptake (de Oliveira et al., 2004; Frizzo et al., 2003; Kovács, Kékesi, Juhász & Dobolyi, 2014; Schmidt et al., 2007; Torres et al., 2010). It has been shown that extracellular guanosine adjusts extracellular adenosine levels (Jackson et al., 2013).

Regarding network communication, the expression of the astrocytic gap junction proteins connexin 30 and 43 is reduced in the BBB‐induced epileptogenic cortex. Carbenoxolone is a broad‐spectrum GJ blocker with additional anti‐inflammatory and mineralocorticoid‐like properties (Li et al., 2019; Nilsen et al., 2006). In vitro and in vivo experiments have shown that carbenoxolone can decrease seizure‐like activities in different types of epilepsy models (Franco‐Pérez et al., 2018; Gigout et al., 2006; Sefil et al., 2012; Ventura‐Mejía & Medina‐Ceja, 2014). For example, blockade of the GJ with carbenoxolone has decreased the duration of seizures and the amplitude of the seizure discharges in 4‐aminopyridine induced epilepsy in anesthetized rats (Gajda et al., 2003). Furthermore, local administration of carbenoxolone in an in vivo model of refractory epilepsy in un‐anesthetized rats also reduced the percentage of seizure time (Nilsen et al., 2006).

Blocking the specific inflammatory pathways which are activated during epileptogenesis may also decrease the severity and the occurrence of spontaneous seizures (Aronica et al., 2012). Microglia and astrocytes are the main sources of IL‐1β in epileptogenic brain tissue and IL‐1R1 is overexpressed in both neurons and glia. Using VX‐765 which is a selective interleukin converting enzyme inhibitor has inhibited the endogenous production of IL‐1β and interfered with the promotion of generalized motor seizures in stimulated rats but not fully kindled rats in a kindling model (T. Ravizza et al., 2008). Furthermore, cannabinoid (CB) receptors, as mediators of endocannabinoid signaling, have demonstrated an immunomodulatory effect on astrocytes (Fields et al., 2020; Sheng et al., 2005). As mentioned in Section 9, TGF‐βR is a putative candidate for albumin uptake and its expression is enhanced following brain insults. TGF‐βR1 kinase activity inhibitor, SB431542, has been reported to prevent the albumin uptake (Ivens et al., 2007; Morganti‐Kossmann et al., 2002). Therefore, targeting the TGF‐βRs may have therapeutic advantages in posttraumatic epilepsy syndromes. There can be other potential targets such as understanding the mechanisms by which GS is regulated which may lead to novel therapeutic approaches to MTLE, the frequently refractory epilepsy to antiepileptic drugs.

## 14 DISCUSSION

As mentioned above, various likely mechanisms are leading to recurrent seizures and epilepsy. Astrocytes are involved in brain inflammation and inflammation is not only the cause but also the consequence of epilepsy, providing a vicious cycle and recurrence of seizures. Also, astrocytes serve as both sources and targets of related inflammatory cell signaling receptors (Aronica et al., 2012). IL‐1β through IL‐1R type 1 receptors increases the extracellular glutamate release by inhibiting astrocytic glutamate reuptake and increasing glial release via induction of TNF‐α, glutamate, in turn, enhances the brain excitability (Bezzi et al., 2001). Inhibition of the production of IL‐1β in astrocytes has been shown to decrease the spike and wave discharges in rats with genetic absence epilepsy (GAERS) (Akin et al., 2011). During the past decade, the key role of astrocytes in the CNS innate immune system has been more elucidated (F. Aloisi et al., 2000; Farina et al., 2007). Astrocyte immune‐inflammatory dysregulation is a common factor in a variety of epilepsy models. Therefore, pharmacological blocking of inflammatory pathways may reduce the intensity and frequency of spontaneous seizures (Aronica et al., 2012). It has been observed that even adjunctive therapy with certain anti‐inflammatory drugs such as statins may exert beneficial effects in SE prognosis in the clinical setting (Vezzani et al., 2015). When the bioelectrical activity of a group of cerebral cortical neurons increases and their activity is hypersynchronized, epileptic seizures occur (Avoli et al., 2005). The gap junctions between adjacent neurons and astrocytes are important ultrastructural elements in hypersynchronization (Sendrowski & Sobaniec, 2013). Therefore, blocking the gap junctions may reduce seizure severity or frequency.

It has been assumed that seizures give rise to seizures (Gillespie, 1902). Accordingly, it has been observed that HS is not only the cause but also the result of drug‐resistant epilepsy (Sendrowski & Sobaniec, 2013). Thus, prevention and treatment of seizures lead to better outcomes and prognoses. Astrocytes are the major type of glia in the CNS with bidirectional flow of information between adjacent cells and a variety of receptors and neurotransmitters so\that targeting astrocytes can be a promising approach in ameliorating epileptic conditions.

## 15 CONCLUSION

As above mentioned, astrocytes are active contributors of ions concentration such as Ca^2+^ and K^+^ in the brain. They are also the main regulators of glutamate and GABA, and exert an important regulatory effect on pH. Considering the roles of the mentioned ions, neurotransmitters, and pH in the neuronal activity in the brain, the roles of astrocytes in epilepsy are conceivable. Astrocytes also have important roles in the function of gap junctions and BBB, immunity, inflammation, and regulation of cytokines. Therefore, it is conceivable that astrocytes play a key role in epilepsy and targeting astrocytes can be a new approach in the treatment or prevention of epilepsy.

## CONFLICT OF INTEREST

There is no conflict of interest.

## AUTHOR CONTRIBUTIONS

Conceptualization and Investigation [HP, RA, HM]; Writing – Original Draft, [all authors participated in the writing]; Writing – Review & Editing, [HP, RA, HM, HV]; Supervision, [HP, RA, HM].

## References

[phy215239-bib-0001] Agostinho, P. , Madeira, D. , Dias, L. , Simões, A. P. , Cunha, R. A. , & Canas, P. M. (2020). Purinergic signaling orchestrating neuron‐glia communication. Pharmacological Research, 162, 105253. 10.1016/j.phrs.2020.105253 33080321

[phy215239-bib-0002] Akin, D. , Ravizza, T. , Maroso, M. , Carcak, N. , Eryigit, T. , Vanzulli, I. , Aker, R. G. , Vezzani, A. , & Onat, F. Y. (2011). IL‐1β is induced in reactive astrocytes in the somatosensory cortex of rats with genetic absence epilepsy at the onset of spike‐and‐wave discharges, and contributes to their occurrence. Neurobiology of Disease, 44(3), 259–269. 10.1016/j.nbd.2011.05.015 21645619

[phy215239-bib-0003] Alemanno, F. (2020). Glutamate–GABA Collateral Cycle Biochemistry for Anesthesiologists and Intensivists (pp. 23–34). Springer.

[phy215239-bib-0004] Aloisi, F. , Ria, F. , & Adorini, L. (2000). Regulation of T‐cell responses by CNS antigen‐presenting cells: Different roles for microglia and astrocytes. Immunology Today, 21(3), 141–147. 10.1016/s0167-5699(99)01512-1 10689302

[phy215239-bib-0005] Aloisi, F. , Ria, F. , Penna, G. , & Adorini, L. (1998). Microglia are more efficient than astrocytes in antigen processing and in Th1 but not Th2 cell activation. The Journal of Immunology, 160(10), 4671–4680. 9590212

[phy215239-bib-0006] Aoki, Y. , Hanai, S. , Sukigara, S. , Otsuki, T. , Saito, T. , Nakagawa, E. , Kaido, T. , Kaneko, Y. , Takahashi, A. , Ikegaya, N. , Iwasaki, M. , Sugai, K. , Sasaki, M. , Goto, Y. , Oka, A. , & Itoh, M. (2019). Altered expression of astrocyte‐related receptors and channels correlates with epileptogenesis in hippocampal sclerosis. Pediatric and Developmental Pathology, 22(6), 532–539. 10.1177/1093526619855488 31166880

[phy215239-bib-0007] Aquilino, M. S. , Whyte‐Fagundes, P. , Zoidl, G. , & Carlen, P. L. (2019). Pannexin‐1 channels in epilepsy. Neuroscience Letters, 695, 71–75. 10.1016/j.neulet.2017.09.004 28886985

[phy215239-bib-0008] Aronica, E. , & Crino, P. B. (2011). Inflammation in epilepsy: Clinical observations. Epilepsia, 52, 26–32. 10.1111/j.1528-1167.2011.03033.x 21542843

[phy215239-bib-0009] Aronica, E. , Ravizza, T. , Zurolo, E. , & Vezzani, A. (2012). Astrocyte immune responses in epilepsy. Glia, 60(8), 1258–1268. 22331574 10.1002/glia.22312

[phy215239-bib-0010] Attwell, D. , Buchan, A. M. , Charpak, S. , Lauritzen, M. , MacVicar, B. A. , & Newman, E. A. (2010). Glial and neuronal control of brain blood flow. Nature, 468(7321), 232–243. 21068832 10.1038/nature09613 PMC3206737

[phy215239-bib-0011] Avoli, M. , Louvel, J. , Pumain, R. , & Köhling, R. (2005). Cellular and molecular mechanisms of epilepsy in the human brain. Progress in Neurobiology, 77(3), 166–200. 10.1016/j.pneurobio.2005.09.006 16307840

[phy215239-bib-0012] Banks, W. A. , Kovac, A. , & Morofuji, Y. (2018). Neurovascular unit crosstalk: Pericytes and astrocytes modify cytokine secretion patterns of brain endothelial cells. Journal of Cerebral Blood Flow & Metabolism, 38(6), 1104–1118. 10.1177/0271678X17740793 29106322 PMC5998993

[phy215239-bib-0013] Bauer, B. , Hartz, A. M. , Pekcec, A. , Toellner, K. , Miller, D. S. , & Potschka, H. (2008). Seizure‐induced up‐regulation of P‐glycoprotein at the blood‐brain barrier through glutamate and cyclooxygenase‐2 signaling. Molecular Pharmacology, 73(5), 1444–1453. 10.1124/mol.107.041210 18094072

[phy215239-bib-0014] Bauer, J. , Elger, C. E. , Hans, V. H. , Schramm, J. , Urbach, H. , Lassmann, H. , & Bien, C. G. (2007). Astrocytes are a specific immunological target in Rasmussen's encephalitis. Annals of Neurology, 62(1), 67–80. 10.1002/ana.21148 17503512

[phy215239-bib-0015] Beghi, E. (2020). The epidemiology of epilepsy. Neuroepidemiology, 54(2), 185–191. 10.1159/000503831 31852003

[phy215239-bib-0016] Benson, M. J. , Manzanero, S. , & Borges, K. (2015). Complex alterations in microglial M1/M2 markers during the development of epilepsy in two mouse models. Epilepsia, 56(6), 895–905. 10.1111/epi.12960 25847097

[phy215239-bib-0017] Bezzi, P. , Domercq, M. , Brambilla, L. , Galli, R. , Schols, D. , De Clercq, E. , Vescovi, A. , Bagetta, G. , Kollias, G. , Meldolesi, J. , & Volterra, A. (2001). CXCR4‐activated astrocyte glutamate release via TNFα: Amplification by microglia triggers neurotoxicity. Nature Neuroscience, 4(7), 702–710. 10.1038/89490 11426226

[phy215239-bib-0018] Binder, D. K. , & Carson, M. J. (2013). Glial cells as primary therapeutic targets for epilepsy. Elsevier. 10.1016/j.neuint.2013.09.004 24021493

[phy215239-bib-0019] Binder, D. K. , & Steinhäuser, C. (2006). Functional changes in astroglial cells in epilepsy. Glia, 54(5), 358–368. 10.1002/glia.20394 16886201

[phy215239-bib-0020] Binder, D. , Yao, X. , Verkman, A. , & Manley, G. (2006). Increased seizure duration in mice lacking aquaporin‐4 water channels Brain Edema XIII (pp. 389–392). Springer. 10.1007/3-211-30714-1_80 16671491

[phy215239-bib-0021] Blanchard, S. , Saillet, S. , Ivanov, A. , Benquet, P. , Bénar, C.‐G. , Pélégrini‐Issac, M. , Benali, H. , & Wendling, F. (2016). A new computational model for neuro‐glio‐vascular coupling: Astrocyte activation can explain cerebral blood flow nonlinear response to interictal events. PLoS One, 11(2), e0147292. 10.1371/journal.pone.0147292 26849643 PMC4743967

[phy215239-bib-0022] Boison, D. , Chen, J.‐F. , & Fredholm, B. B. (2010). Adenosine signaling and function in glial cells. Cell Death & Differentiation, 17(7), 1071–1082. 10.1038/cdd.2009.131 19763139 PMC2885470

[phy215239-bib-0023] Bonifati, D. M. , & Kishore, U. (2007). Role of complement in neurodegeneration and neuroinflammation. Molecular Immunology, 44(5), 999–1010. 10.1016/j.molimm.2006.03.007 16698083

[phy215239-bib-0024] Bordey, A. , & Spencer, D. (2004). Distinct electrophysiological alterations in dentate gyrus versus CA1 glial cells from epileptic humans with temporal lobe sclerosis. Epilepsy Research, 59(2–3), 107–122. 10.1016/j.eplepsyres.2004.04.004 15246115

[phy215239-bib-0025] Chan, F. , Lax, N. Z. , Voss, C. M. , Aldana, B. I. , Whyte, S. , Jenkins, A. , Nicholson, C. , Nichols, S. , Tilley, E. , Powell, Z. , Waagepetersen, H. S. , Davies, C. H. , Turnbull, D. M. , & Cunningham, M. O. (2019). The role of astrocytes in seizure generation: Insights from a novel in vitro seizure model based on mitochondrial dysfunction. Brain, 142(2), 391–411. 10.1093/brain/awy320 30689758 PMC6519661

[phy215239-bib-0026] Chung, S. , Guo, F. , Jiang, P. , Pleasure, D. E. , & Deng, W. (2013). Olig2/Plp‐positive progenitor cells give rise to Bergmann glia in the cerebellum. Cell Death & Disease, 4(3), e546. 10.1038/cddis.2013.74 23492777 PMC3615735

[phy215239-bib-0027] Chu‐Shore, C. J. , Major, P. , Camposano, S. , Muzykewicz, D. , & Thiele, E. A. (2010). The natural history of epilepsy in tuberous sclerosis complex. Epilepsia, 51(7), 1236–1241. 10.1111/j.1528-1167.2009.02474.x 20041940 PMC3065368

[phy215239-bib-0028] Collignon, F. , Wetjen, N. M. , Cohen‐Gadol, A. A. , Cascino, G. D. , Parisi, J. , Meyer, F. B. , Marsh, W. R. , Roche, P. , & Weigand, S. D. (2006). Altered expression of connexin subtypes in mesial temporal lobe epilepsy in humans. Journal of Neurosurgery, 105(1), 77–87. 10.3171/jns.2006.105.1.77 16874892

[phy215239-bib-0029] Curatolo, P. , Nabbout, R. , Lagae, L. , Aronica, E. , Ferreira, J. C. , Feucht, M. , Hertzberg, C. , Jansen, A. C. , Jansen, F. , Kotulska, K. , Moavero, R. , O'Callaghan, F. , Papavasiliou, A. , Tzadok, M. , & Jóźwiak, S. (2018). Management of epilepsy associated with tuberous sclerosis complex: Updated clinical recommendations. European Journal of Paediatric Neurology, 22(5), 738–748. 10.1016/j.ejpn.2018.05.006 29880258

[phy215239-bib-0030] Dadas, A. , & Janigro, D. (2019). Breakdown of blood brain barrier as a mechanism of post‐traumatic epilepsy. Neurobiology of Disease, 123, 20–26. 10.1016/j.nbd.2018.06.022 30030025 PMC6794150

[phy215239-bib-0031] D'Alimonte, I. , D'Auro, M. , Citraro, R. , Biagioni, F. , Jiang, S. , Nargi, E. , & Ciccarelli, R. (2009). Altered distribution and function of A2A adenosine receptors in the brain of WAG/Rij rats with genetic absence epilepsy, before and after appearance of the disease. European Journal of Neuroscience, 30(6), 1023–1035. 10.1111/j.1460-9568.2009.06897.x 19723291

[phy215239-bib-0032] David, Y. , Cacheaux, L. P. , Ivens, S. , Lapilover, E. , Heinemann, U. , Kaufer, D. , & Friedman, A. (2009). Astrocytic dysfunction in epileptogenesis: Consequence of altered potassium and glutamate homeostasis? Journal of Neuroscience, 29(34), 10588–10599. 10.1523/JNEUROSCI.2323-09.2009 19710312 PMC2875068

[phy215239-bib-0033] de Oliveira, D. L. , Horn, J. F. , Rodrigues, J. M. , Frizzo, M. E. , Moriguchi, E. , Souza, D. O. , & Wofchuk, S. (2004). Quinolinic acid promotes seizures and decreases glutamate uptake in young rats: Reversal by orally administered guanosine. Brain Research, 1018(1), 48–54. 10.1016/j.brainres.2004.05.033 15262204

[phy215239-bib-0034] De Simoni, M. G. , Perego, C. , Ravizza, T. , Moneta, D. , Conti, M. , Marchesi, F. , De Luigi, A. , Garattini, S. , & Vezzani, A. (2000). Inflammatory cytokines and related genes are induced in the rat hippocampus by limbic status epilepticus. European Journal of Neuroscience, 12(7), 2623–2633. 10.1046/j.1460-9568.2000.00140.x 10947836

[phy215239-bib-0035] Dias, R. B. , Rombo, D. M. , Ribeiro, J. A. , Henley, J. M. , & Sebastião, A. M. (2013). Adenosine: Setting the stage for plasticity. Trends in Neurosciences, 36(4), 248–257. 10.1016/j.tins.2012.12.003 23332692

[phy215239-bib-0036] Dube, C. M. , Ravizza, T. , Hamamura, M. , Zha, Q. , Keebaugh, A. , Fok, K. , Andres, A. L. , Nalcioglu, O. , Obenaus, A. , Vezzani, A. , & Baram, T. Z. (2010). Epileptogenesis provoked by prolonged experimental febrile seizures: Mechanisms and biomarkers. Journal of Neuroscience, 30(22), 7484–7494. 10.1523/JNEUROSCI.0551-10.2010 20519523 PMC2906240

[phy215239-bib-0037] Eid, T. , Tu, N. , Lee, T.‐S.‐ W. , & Lai, J. C. (2013). Regulation of astrocyte glutamine synthetase in epilepsy. Neurochemistry International, 63(7), 670–681. 10.1016/j.neuint.2013.06.008 23791709 PMC3825815

[phy215239-bib-0038] Ekonomou, A. , Angelatou, F. , Vergnes, M. , & Kostopoulos, G. (1998). Lower density of A1 adenosine receptors in nucleus reticularis thalami in rats with genetic absence epilepsy. NeuroReport, 9(9), 2135–2140. 10.1097/00001756-199806220-00042 9674608

[phy215239-bib-0039] Ekonomou, A. , Sperk, G. , Kostopoulos, G. , & Angelatou, F. (2000). Reduction of A1 adenosine receptors in rat hippocampus after kainic acid‐induced limbic seizures. Neuroscience Letters, 284(1–2), 49–52. 10.1016/S0304-3940(00)00954-X 10771159

[phy215239-bib-0040] Elisevich, K. , Rempel, S. A. , Smith, B. J. , & Edvardsen, K. (1997). Hippocampal connexin 43 expression in human complex partial seizure disorder. Experimental Neurology, 145(1), 154–164. 10.1006/exnr.1997.6467 9184118

[phy215239-bib-0041] Engel, J. Jr , & Pitkänen, A. (2020). Biomarkers for epileptogenesis and its treatment. Neuropharmacology, 167, 107735. 31377200 10.1016/j.neuropharm.2019.107735 PMC6994353

[phy215239-bib-0042] Engel, T. , & Henshall, D. C. (2009). Apoptosis, Bcl‐2 family proteins and caspases: The ABCs of seizure‐damage and epileptogenesis? International Journal of Physiology, Pathophysiology and Pharmacology, 1(2), 97. PMC3047241 21383882

[phy215239-bib-0043] Farina, C. , Aloisi, F. , & Meinl, E. (2007). Astrocytes are active players in cerebral innate immunity. Trends in Immunology, 28(3), 138–145. 10.1016/j.it.2007.01.005 17276138

[phy215239-bib-0044] Fellin, T. , & Haydon, P. G. (2005). Do astrocytes contribute to excitation underlying seizures? Trends in Molecular Medicine, 11(12), 530–533. 10.1016/j.molmed.2005.10.007 16290019

[phy215239-bib-0045] Fields, J. A. , Swinton, M. K. , Montilla‐Perez, P. , Ricciardelli, E. , & Telese, F. (2020). The cannabinoid receptor agonist, WIN, suppresses the activation of proinflammatory genes induced by interleukin 1 beta in human astrocytes. Cannabis and Cannabinoid Research, 7(1), 78–92. 10.1089/can.2020.0128 33998879 PMC8864424

[phy215239-bib-0046] Fonseca, C. G. , Green, C. R. , & Nicholson, L. F. (2002). Upregulation in astrocytic connexin 43 gap junction levels may exacerbate generalized seizures in mesial temporal lobe epilepsy. Brain Research, 929(1), 105–116. 10.1016/S0006-8993(01)03289-9 11852037

[phy215239-bib-0047] Foresti, M. L. , Arisi, G. M. , & Shapiro, L. A. (2011). Role of glia in epilepsy‐associated neuropathology, neuroinflammation and neurogenesis. Brain Research Reviews, 66(1–2), 115–122. 10.1016/j.brainresrev.2010.09.002 20837059

[phy215239-bib-0048] Franco‐Pérez, J. , Manjarrez‐Marmolejo, J. , Rodríguez‐Balderas, C. , Castro, N. , & Ballesteros‐Zebadua, P. (2018). Quinine and carbenoxolone enhance the anticonvulsant activity of some classical antiepileptic drugs. Neurological Research, 40(1), 26–33. 10.1080/01616412.2017.1384092 28988516

[phy215239-bib-0049] Fredholm, B. B. (2012). Rethinking the purinergic neuron–glia connection. Proceedings of the National Academy of Sciences, 109(16), 5913–5914. 10.1073/pnas.1203764109 PMC3340997 22509008

[phy215239-bib-0050] Frizzo, M. E. , Antunes Soares, F. A. , Dall'Onder, L. P. , Lara, D. R. , Swanson, R. A. , & Souza, D. O. (2003). Extracellular conversion of guanine‐based purines to guanosine specifically enhances astrocyte glutamate uptake. Brain Research, 972(1–2), 84–89. 10.1016/s0006-8993(03)02506-x 12711081

[phy215239-bib-0051] Gajda, Z. , Gyengési, E. , Hermesz, E. , Ali, K. S. , & Szente, M. (2003). Involvement of gap junctions in the manifestation and control of the duration of seizures in rats in vivo. Epilepsia, 44(12), 1596–1600. 10.1111/j.0013-9580.2003.25803.x 14636335

[phy215239-bib-0052] Galic, M. A. , Riazi, K. , & Pittman, Q. J. (2012). Cytokines and brain excitability. Frontiers in Neuroendocrinology, 33(1), 116–125. 10.1016/j.yfrne.2011.12.002 22214786 PMC3547977

[phy215239-bib-0053] Gigout, S. , Louvel, J. , & Pumain, R. (2006). Effects in vitro and in vivo of a gap junction blocker on epileptiform activities in a genetic model of absence epilepsy. Epilepsy Research, 69(1), 15–29. 10.1016/j.eplepsyres.2005.12.002 16466906

[phy215239-bib-0054] Gillespie, A. L. (1902). Epilepsy and other chronic convulsive diseases: Their causes, symptoms, and treatment. Edinburgh Medical Journal, 11(3), 271.

[phy215239-bib-0055] Giovannoni, F. , & Quintana, F. J. (2020). The role of astrocytes in CNS inflammation. Trends in Immunology, 41(9), 805–819. 32800705 10.1016/j.it.2020.07.007 PMC8284746

[phy215239-bib-0056] Glass, M. , Faull, R. , Bullock, J. Y. , Jansen, K. , Mee, E. W. , Walker, E. B. , Synek, B. , & Dragunow, M. (1996). Loss of A1 adenosine receptors in human temporal lobe epilepsy. Brain Research, 710(1–2), 56–68. 10.1016/0006-8993(95)01313-X 8963679

[phy215239-bib-0057] Gómez‐Gonzalo, M. , Losi, G. , Chiavegato, A. , Zonta, M. , Cammarota, M. , Brondi, M. , Vetri, F. , Uva, L. , Pozzan, T. , de Curtis, M. , Ratto, G. M. , & Carmignoto, G. (2010). An excitatory loop with astrocytes contributes to drive neurons to seizure threshold. PLoS Biology, 8(4), e1000352. 10.1371/journal.pbio.1000352 20405049 PMC2854117

[phy215239-bib-0058] Griffiths, M. R. , Neal, J. W. , Fontaine, M. , Das, T. , & Gasque, P. (2009). Complement factor H, a marker of self protects against experimental autoimmune encephalomyelitis. The Journal of Immunology, 182(7), 4368–4377. 10.4049/jimmunol.0800205 19299737

[phy215239-bib-0059] Gupta, A. , de Bruyn, G. , Tousseyn, S. , Krishnan, B. , Lagae, L. , Agarwal, N. , Minnesota Epilepsy Group , Frost, M. , Sparagana, S. , LaJoie, J. , Riviello, J. , Devinsky, O. , LaJoie, J. , Thiele, E. , McClintock, W. , Kohrman, M. , Brown, C. , Kuperman, R. , Wu, J. , … Jeong, A. (2020). Epilepsy and neurodevelopmental comorbidities in tuberous sclerosis complex: A natural history study. Pediatric Neurology, 106, 10–16. 10.1016/j.pediatrneurol.2019.12.016 32139167

[phy215239-bib-0060] Hajishengallis, G. , & Lambris, J. D. (2010). Crosstalk pathways between Toll‐like receptors and the complement system. Trends in Immunology, 31(4), 154–163. 10.1016/j.it.2010.01.002 20153254 PMC2849859

[phy215239-bib-0061] Heinemann, U. , Kaufer, D. , & Friedman, A. (2012). Blood‐brain barrier dysfunction, TGFβ signaling, and astrocyte dysfunction in epilepsy. Glia, 60(8), 1251–1257. 22378298 10.1002/glia.22311 PMC3615248

[phy215239-bib-0062] Heithoff, B. P. , George, K. K. , Phares, A. N. , Zuidhoek, I. A. , Munoz‐Ballester, C. , & Robel, S. (2021). Astrocytes are necessary for blood–brain barrier maintenance in the adult mouse brain. Glia, 69(2), 436–472. 10.1002/glia.23908 32955153 PMC7736206

[phy215239-bib-0063] Héja, L. , Nyitrai, G. , Kékesi, O. , Dobolyi, Á. , Szabó, P. , Fiáth, R. , Ulbert, I. , Pál‐Szenthe, B. , Palkovits, M. , & Kardos, J. (2012). Astrocytes convert network excitation to tonic inhibition of neurons. BMC Biology, 10(1), 1–21. 10.1186/1741-7007-10-26 22420899 PMC3342137

[phy215239-bib-0064] Hiragi, T. , Ikegaya, Y. , & Koyama, R. (2018). Microglia after seizures and in epilepsy. Cells, 7(4), 26. 10.3390/cells7040026 PMC5946103 29597334

[phy215239-bib-0065] Hyun, H.‐W. , Min, S.‐J. , & Kim, J.‐E. (2017). CDK5 inhibitors prevent astroglial apoptosis and reactive astrogliosis by regulating PKA and DRP1 phosphorylations in the rat hippocampus. Neuroscience Research, 119, 24–37. 10.1016/j.neures.2017.01.006 28153522

[phy215239-bib-0066] Ivens, S. , Kaufer, D. , Flores, L. P. , Bechmann, I. , Zumsteg, D. , Tomkins, O. , & Friedman, A. (2007). TGF‐beta receptor‐mediated albumin uptake into astrocytes is involved in neocortical epileptogenesis. Brain, 130(Pt 2), 535–547. 10.1093/brain/awl317 17121744

[phy215239-bib-0067] Jackson, E. K. , Cheng, D. , Jackson, T. C. , Verrier, J. D. , & Gillespie, D. G. (2013). Extracellular guanosine regulates extracellular adenosine levels. American Journal of Physiology‐Cell Physiology, 304(5), C406–C421. 10.1152/ajpcell.00212.2012 23242185 PMC3602643

[phy215239-bib-0068] Jennings, L. L. , Hao, C. , Cabrita, M. A. , Vickers, M. F. , Baldwin, S. A. , Young, J. D. , & Cass, C. E. (2001). Distinct regional distribution of human equilibrative nucleoside transporter proteins 1 and 2 (hENT1 and hENT2) in the central nervous system. Neuropharmacology, 40(5), 722–731. 10.1016/S0028-3908(00)00207-0 11311901

[phy215239-bib-0069] Kadry, H. , Noorani, B. , & Cucullo, L. (2020). A blood–brain barrier overview on structure, function, impairment, and biomarkers of integrity. Fluids and Barriers of the CNS, 17(1), 1–24. 10.1186/s12987-020-00230-3 33208141 PMC7672931

[phy215239-bib-0070] Kan, M. , Song, L. , Zhang, X. , Zhang, J. , & Fang, P. (2019). Circulating high mobility group box‐1 and toll‐like receptor 4 expressions increase the risk and severity of epilepsy. Brazilian Journal of Medical and Biological Research, 52. 10.1590/1414-431x20197374 PMC6596364 31241711

[phy215239-bib-0071] Kinboshi, M. , Ikeda, A. , & Ohno, Y. (2020). Role of astrocytic inwardly rectifying potassium (Kir) 4.1 channels in epileptogenesis. Frontiers in Neurology, 11, 1832. 10.3389/fneur.2020.626658 PMC7786246 33424762

[phy215239-bib-0072] Ko, A.‐R. , Hyun, H.‐W. , Min, S.‐J. , & Kim, J.‐E. (2016). The differential DRP1 phosphorylation and mitochondrial dynamics in the regional specific astroglial death induced by status epilepticus. Frontiers in Cellular Neuroscience, 10, 124. 10.3389/fncel.2016.00124 27242436 PMC4870264

[phy215239-bib-0073] Kong, Q. , Takahashi, K. , Schulte, D. , Stouffer, N. , Lin, Y. , & Lin, C. L. (2012). Increased glial glutamate transporter EAAT2 expression reduces epileptogenic processes following pilocarpine‐induced status epilepticus. Neurobiology of Diseases, 47(2), 145–154. 10.1016/j.nbd.2012.03.032 PMC3572547 22513140

[phy215239-bib-0074] Kovács, Z. , Juhász, G. , Palkovits, M. , Dobolyi, Á ., & Kekesi, K. A. (2011). Area, age and gender dependence of the nucleoside system in the brain: A review of current literature. Current Topics in Medicinal Chemistry, 11(8), 1012–1033. 21401498 10.2174/156802611795347636

[phy215239-bib-0075] Kovács, Z. , Kardos, J. , Kekesi, K. A. , Juhász, G. , Lakatos, R. , & Héja, L. (2015). Effects of nucleosides on glia‐neuron interactions open up new vistas in the development of more effective antiepileptic drugs. Current Medicinal Chemistry, 22(12), 1500–1514. 25666791 10.2174/0929867322666150212153210

[phy215239-bib-0076] Kovács, Z. , Kekesi, K. A. , Juhász, G. , Barna, J. , Héja, L. , Lakatos, R. , & Dobolyi, Á. (2014). Non‐adenosine nucleoside inosine, guanosine and uridine as promising antiepileptic drugs: A summary of current literature. Mini Reviews in Medicinal Chemistry, 14(13), 1033–1042. 10.2174/1389557514666141107120226 25382017

[phy215239-bib-0077] Kovács, Z. , Kékesi, K. , Juhász, G. , & Dobolyi, A. (2014). The antiepileptic potential of nucleosides. Current Medicinal Chemistry, 21(6), 788–821. 24251559 10.2174/1381612819666131119154505

[phy215239-bib-0078] Kumar, V. (2019). The complement system, toll‐like receptors and inflammasomes in host defense: Three musketeers’ one target: The CS, TLRs, and inflammasomes are the first line of immune defense working by crosstalking with each other to mount the effective immune response. International Reviews of Immunology, 38(4), 131–156. 10.1080/08830185.2019.1609962 31066339

[phy215239-bib-0079] Kwon, H. S. , & Koh, S.‐H. (2020). Neuroinflammation in neurodegenerative disorders: The roles of microglia and astrocytes. Translational Neurodegeneration, 9(1), 1–12. 10.1186/s40035-020-00221-2 33239064 PMC7689983

[phy215239-bib-0080] Lee, D. A. , Lee, H. J. , Kim, H. C. , & Park, K. M. (2021). Temporal lobe epilepsy with or without hippocampal sclerosis: Structural and functional connectivity using advanced MRI techniques. Journal of Neuroimaging. 10.1111/jon.12898 34110654

[phy215239-bib-0081] Lee, T.‐S. , Mane, S. , Eid, T. , Zhao, H. , Lin, A. , Guan, Z. , Kim, J. H. , Schweitzer, J. , King‐Stevens, D. , Weber, P. , Spencer, S. S. , Spencer, D. D. , & de Lanerolle, N. C. (2007). Gene expression in temporal lobe epilepsy is consistent with increased release of glutamate by astrocytes. Molecular Medicine, 13(1), 1–13. 10.2119/2006-00079.Lee 17515952 PMC1869627

[phy215239-bib-0082] Li, Q. , Li, Q. Q. , Jia, J. N. , Liu, Z. Q. , Zhou, H. H. , & Mao, X. Y. (2019). Targeting gap junction in epilepsy: Perspectives and challenges. Biomedicine & Pharmacotherapy, 109, 57–65. 10.1016/j.biopha.2018.10.068 30396092

[phy215239-bib-0083] Magnotti, L. M. , Goodenough, D. A. , & Paul, D. L. (2011). Deletion of oligodendrocyte Cx32 and astrocyte Cx43 causes white matter vacuolation, astrocyte loss and early mortality. Glia, 59(7), 1064–1074. 10.1002/glia.21179 21538560 PMC3094483

[phy215239-bib-0084] Mantegazza, Renato , Bernasconi, Pia , Baggi, Fulvio , Spreafico, Roberto , Ragona, Francesca , Antozzi, Carlo , Bernardi, Gaetano , Granata, Tiziana , & Group, I. R. s. E. S . (2002). Antibodies against GluR3 peptides are not specific for Rasmussen's encephalitis but are also present in epilepsy patients with severe, early onset disease and intractable seizures. Journal of Neuroimmunology, 131(1–2), 179–185. 10.1016/S0165-5728(02)00261-8 12458050

[phy215239-bib-0085] Marina, N. , Christie, I. N. , Korsak, A. , Doronin, M. , Brazhe, A. , Hosford, P. S. , Wells, J. A. , Sheikhbahaei, S. , Humoud, I. , Paton, J. F. R. , Lythgoe, M. F. , Semyanov, A. , Kasparov, S. , & Gourine, A. V. (2020). Astrocytes monitor cerebral perfusion and control systemic circulation to maintain brain blood flow. Nature Communications, 11(1), 1–9. 10.1038/s41467-019-13956-y PMC6952443 31919423

[phy215239-bib-0086] Maroso, M. , Balosso, S. , Ravizza, T. , Liu, J. , Aronica, E. , Iyer, A. M. , Rossetti, C. , Molteni, M. , Casalgrandi, M. , Manfredi, A. A. , Bianchi, M. E. , & Vezzani, A. (2010). Toll‐like receptor 4 and high‐mobility group box‐1 are involved in ictogenesis and can be targeted to reduce seizures. Nature Medicine, 16(4), 413–419. 10.1038/nm.2127 20348922

[phy215239-bib-0087] Matejuk, A. , & Ransohoff, R. M. (2020). Crosstalk between astrocytes and microglia: An overview. Frontiers in Immunology, 11, 1416. 10.3389/fimmu.2020.01416 32765501 PMC7378357

[phy215239-bib-0088] Mazaud, D. , Kottler, B. , Gonçalves‐Pimentel, C. , Proelss, S. , Tüchler, N. , Deneubourg, C. , & Lai, E. C. (2019). Transcriptional regulation of the Glutamate/GABA/Glutamine cycle in adult glia controls motor activity and seizures in Drosophila. Journal of Neuroscience, 39(27), 5269–5283. 31064860 10.1523/JNEUROSCI.1833-18.2019 PMC6607755

[phy215239-bib-0089] Md, I. B. , MRCPath, M. T. , & Wiestler, O. D. (2002). Ammon's horn sclerosis: A maldevelopmental disorder associated with temporal lobe epilepsy. Brain Pathology, 12(2), 199–211. 10.1111/j.1750-3639.2002.tb00436.x 11958375 PMC8095862

[phy215239-bib-0090] Mederos, S. , González‐Arias, C. , & Perea, G. (2018). Astrocyte–neuron networks: A multilane highway of signaling for homeostatic brain function. Frontiers in Synaptic Neuroscience, 10, 45. 10.3389/fnsyn.2018.00045 30542276 PMC6277918

[phy215239-bib-0091] Morganti‐Kossmann, M. C. , Rancan, M. , Stahel, P. F. , & Kossmann, T. (2002). Inflammatory response in acute traumatic brain injury: A double‐edged sword. Current Opinion in Critical Care, 8(2), 101–105. 10.1097/00075198-200204000-00002 12386508

[phy215239-bib-0092] Morin‐Brureau, M. , Lebrun, A. , Rousset, M.‐C. , Fagni, L. , Bockaert, J. , de Bock, F. , & Lerner‐Natoli, M. (2011). Epileptiform activity induces vascular remodeling and zonula occludens 1 downregulation in organotypic hippocampal cultures: Role of VEGF signaling pathways. Journal of Neuroscience, 31(29), 10677–10688. 10.1523/JNEUROSCI.5692-10.2011 21775611 PMC6622643

[phy215239-bib-0093] Morotti, F. , Barreiros, T. , Machado, F. , González, S. , Marinho, L. , & Seneda, M. (2018). Is the number of antral follicles an interesting selection criterium for fertility in cattle? Animal Reproduction (AR), 12(3), 479–486.

[phy215239-bib-0094] Naus, C. C. , Bechberger, J. F. , & Paul, D. L. (1991). Gap junction gene expression in human seizure disorder. Experimental Neurology, 111(2), 198–203. 10.1016/0014-4886(91)90007-Y 1846600

[phy215239-bib-0095] Nickels, K. C. , & Noe, K. (2021). Etiology and pathology of epilepsy. Epilepsy, 23–35.

[phy215239-bib-0096] Nilsen, K. E. , Kelso, A. R. , & Cock, H. R. (2006). Antiepileptic effect of gap‐junction blockers in a rat model of refractory focal cortical epilepsy. Epilepsia, 47(7), 1169–1175. 10.1111/j.1528-1167.2006.00540.x 16886980

[phy215239-bib-0097] Onodera, M. , Meyer, J. , Furukawa, K. , Hiraoka, Y. , Aida, T. , Tanaka, K. , Tanaka, K. F. , Rose, C. R. , & Matsui, K. O. (2021). Exacerbation of epilepsy by astrocyte alkalization and gap junction uncoupling. Journal of Neuroscience, 41(10), 2106–2118. 10.1523/JNEUROSCI.2365-20.2020 33478985 PMC8018766

[phy215239-bib-0098] Parker, W. E. , Orlova, K. A. , Heuer, G. G. , Baybis, M. , Aronica, E. , Frost, M. , Wong, M. , & Crino, P. B. (2011). Enhanced epidermal growth factor, hepatocyte growth factor, and vascular endothelial growth factor expression in tuberous sclerosis complex. The American Journal of Pathology, 178(1), 296–305. 10.1016/j.ajpath.2010.11.031 21224066 PMC3069836

[phy215239-bib-0099] Patel, D. C. , Tewari, B. P. , Chaunsali, L. , & Sontheimer, H. (2019). Neuron–glia interactions in the pathophysiology of epilepsy. Nature Reviews Neuroscience, 20(5), 282–297. 10.1038/s41583-019-0126-4 30792501 PMC8558781

[phy215239-bib-0100] Patterson, K. P. , Brennan, G. P. , Curran, M. , Kinney‐Lang, E. , Dubé, C. , Rashid, F. , Ly, C. , Obenaus, A. , & Baram, T. Z. (2015). Rapid, coordinate inflammatory responses after experimental febrile status epilepticus: Implications for epileptogenesis. Eneuro, 2(5). 10.1523/ENEURO.0034-15.2015 PMC4699830 26730400

[phy215239-bib-0101] Perea, G. , & Araque, A. (2007). Astrocytes potentiate transmitter release at single hippocampal synapses. Science, 317(5841), 1083–1086. 17717185 10.1126/science.1144640

[phy215239-bib-0102] Raimondo, J. V. , Burman, R. J. , Katz, A. A. , & Akerman, C. J. (2015). Ion dynamics during seizures. Frontiers in Cellular Neuroscience, 9, 419. 10.3389/fncel.2015.00419 26539081 PMC4612498

[phy215239-bib-0103] Raimondo, J. V. , Tomes, H. , Irkle, A. , Kay, L. , Kellaway, L. , Markram, H. , Millar, R. P. , & Akerman, C. J. (2016). Tight coupling of astrocyte pH dynamics to epileptiform activity revealed by genetically encoded pH sensors. Journal of Neuroscience, 36(26), 7002–7013. 10.1523/JNEUROSCI.0664-16.2016 27358457 PMC5026294

[phy215239-bib-0104] Rajabian, A. , Rajabian, F. , Babaei, F. , Mirzababaei, M. , Nassiri‐Asl, M. , & Hosseinzadeh, H. (2022). Interaction of medicinal plants and their active constituents with potassium ion channels: A systematic review. Frontiers in Pharmacology, 13, 10.3389/fphar.2022.831963 PMC8902679 35273505

[phy215239-bib-0105] Ravizza, T. , Lucas, S.‐M. , Balosso, S. , Bernardino, L. , Ku, G. , Noe, F. , Malva, J. , Randle, J. C. R. , Allan, S. , & Vezzani, A. (2006). Inactivation of caspase‐1 in rodent brain: A novel anticonvulsive strategy. Epilepsia, 47(7), 1160–1168. 10.1111/j.1528-1167.2006.00590.x 16886979

[phy215239-bib-0106] Ravizza, T. , Noé, F. , Zardoni, D. , Vaghi, V. , Sifringer, M. , & Vezzani, A. (2008). Interleukin converting enzyme inhibition impairs kindling epileptogenesis in rats by blocking astrocytic IL‐1beta production. Neurobiology of Diseases, 31(3), 327–333. 10.1016/j.nbd.2008.05.007 18632279

[phy215239-bib-0107] Revuelta, M. , Castaño, A. , Machado, A. , Cano, J. , & Venero, J. L. (2005). Kainate‐induced zinc translocation from presynaptic terminals causes neuronal and astroglial cell death and mRNA loss of BDNF receptors in the hippocampal formation and amygdala. Journal of Neuroscience Research, 82(2), 184–195. 10.1002/jnr.20632 16175575

[phy215239-bib-0108] Riazi, K. , Galic, M. A. , & Pittman, Q. J. (2010). Contributions of peripheral inflammation to seizure susceptibility: Cytokines and brain excitability. Epilepsy Research, 89(1), 34–42. 10.1016/j.eplepsyres.2009.09.004 19804959

[phy215239-bib-0109] Rigau, V. , Morin, M. , Rousset, M.‐C. , de Bock, F. , Lebrun, A. , Coubes, P. , Picot, M.‐C. , Baldy‐Moulinier, M. , Bockaert, J. , Crespel, A. , & Lerner‐Natoli, M. (2007). Angiogenesis is associated with blood–brain barrier permeability in temporal lobe epilepsy. Brain, 130(7), 1942–1956. 10.1093/brain/awm118 17533168

[phy215239-bib-0110] Robel, S. , & Sontheimer, H. (2016). Glia as drivers of abnormal neuronal activity. Nature Neuroscience, 19(1), 28–33. 10.1038/nn.4184 26713746 PMC4966160

[phy215239-bib-0111] Rouach, N. , Koulakoff, A. , Abudara, V. , Willecke, K. , & Giaume, C. (2008). Astroglial metabolic networks sustain hippocampal synaptic transmission. Science, 322(5907), 1551–1555. 19056987 10.1126/science.1164022

[phy215239-bib-0112] Ryu, H. J. , Kim, J.‐E. , Yeo, S.‐I. , & Kang, T.‐C. (2011). p65/RelA‐Ser529 NF‐κB subunit phosphorylation induces autophagic astroglial death (Clasmatodendrosis) following status epilepticus. Cellular and Molecular Neurobiology, 31(7), 1071–1078. 10.1007/s10571-011-9706-1 21598036 PMC11498587

[phy215239-bib-0113] Sakai, K. , Takata, F. , Dohgu, S. , Koga, M. , Kimura, I. , Yamauchi, A. , & Kataoka, Y. (2018). Dysregulation of the CNS supporting vascular and glial cells induces the late posttraumatic epilepsy in mice with mild traumatic brain injury. Paper presented at the Proceedings for Annual Meeting of The Japanese Pharmacological Society WCP2018 (The 18th World Congress of Basic and Clinical Pharmacology).

[phy215239-bib-0114] Schmidt, A. P. , Lara, D. R. , & Souza, D. O. (2007). Proposal of a guanine‐based purinergic system in the mammalian central nervous system. Pharmacology & Therapeutics, 116(3), 401–416. 10.1016/j.pharmthera.2007.07.004 17884172

[phy215239-bib-0115] Schoch, H. J. , Fischer, S. , & Marti, H. H. (2002). Hypoxia‐induced vascular endothelial growth factor expression causes vascular leakage in the brain. Brain, 125(11), 2549–2557. 10.1093/brain/awf257 12390979

[phy215239-bib-0116] Sefil, F. , Bagirici, F. , Acar, M. D. , & Marangoz, C. (2012). Influence of carbenoxolone on the anticonvulsant efficacy of phenytoin in pentylenetetrazole kindled rats. Acta Neurobiologiae Experimentalis, 72(2), 177–184. 22810219 10.55782/ane-2012-1890

[phy215239-bib-0117] Seifert, G. , Carmignoto, G. , & Steinhäuser, C. (2010). Astrocyte dysfunction in epilepsy. Brain Research Reviews, 63(1–2), 212–221. 10.1016/j.brainresrev.2009.10.004 19883685

[phy215239-bib-0118] Seiffert, E. , Dreier, J. P. , Ivens, S. , Bechmann, I. , Tomkins, O. , Heinemann, U. , & Friedman, A. (2004). Lasting blood‐brain barrier disruption induces epileptic focus in the rat somatosensory cortex. Journal of Neuroscience, 24(36), 7829–7836. 10.1523/JNEUROSCI.1751-04.2004 15356194 PMC6729929

[phy215239-bib-0119] Sendrowski, K. , & Sobaniec, W. (2013). Hippocampus, hippocampal sclerosis and epilepsy. Pharmacological Reports, 65(3), 555–565. 10.1016/S1734-1140(13)71033-8 23950578

[phy215239-bib-0120] Sheng, W. , Hu, S. , min, X. , Cabral, G. A. , Lokensgard, J. R. , & Peterson, P. (2005). Synthetic cannabinoid WIN55, 212–2 inhibits generation of inflammatory mediators by IL‐1beta stimulated human astrocytes. Glia, 49, 211–219. 15390091 10.1002/glia.20108

[phy215239-bib-0121] Sofroniew, M. (2014). Astrogliosis. Cold Spring Harbor Perspectives in Biology, 7, a020420. 25380660 10.1101/cshperspect.a020420 PMC4315924

[phy215239-bib-0122] Sosunov, A. A. , Wu, X. , Weiner, H. L. , Mikell, C. B. , Goodman, R. R. , Crino, P. D. , & McKhann, G. M. (2008). Tuberous sclerosis: A primary pathology of astrocytes? Epilepsia, 49, 53–62. 10.1111/j.1528-1167.2008.01493.x 18226172

[phy215239-bib-0123] Sperlágh, B. , & Sylvester Vizi, E. (2011). The role of extracellular adenosine in chemical neurotransmission in the hippocampus and Basal Ganglia: Pharmacological and clinical aspects. Current Topics in Medicinal Chemistry, 11(8), 1034–1046. 21401497 10.2174/156802611795347564 PMC3179034

[phy215239-bib-0124] Strohschein, S. , Hüttmann, K. , Gabriel, S. , Binder, D. K. , Heinemann, U. , & Steinhäuser, C. (2011). Impact of aquaporin‐4 channels on K+ buffering and gap junction coupling in the hippocampus. Glia, 59(6), 973–980. 10.1002/glia.21169 21446052

[phy215239-bib-0125] Swissa, E. , Serlin, Y. , Vazana, U. , Prager, O. , & Friedman, A. (2019). Blood–brain barrier dysfunction in status epileptics: Mechanisms and role in epileptogenesis. Epilepsy & Behavior, 101, 106285. 10.1016/j.yebeh.2019.04.038 31711869

[phy215239-bib-0126] Talos, D. M. , Kwiatkowski, D. J. , Cordero, K. , Black, P. M. , & Jensen, F. E. (2008). Cell‐specific alterations of glutamate receptor expression in tuberous sclerosis complex cortical tubers. Annals of Neurology: Official Journal of the American Neurological Association and the Child Neurology Society, 63(4), 454–465. 10.1002/ana.21342 PMC2701384 18350576

[phy215239-bib-0127] Thanabalasundaram, G. , Pieper, C. , Lischper, M. , & Galla, H.‐J. (2010). Regulation of the blood–brain barrier integrity by pericytes via matrix metalloproteinases mediated activation of vascular endothelial growth factor in vitro. Brain Research, 1347, 1–10. 10.1016/j.brainres.2010.05.096 20553880

[phy215239-bib-0128] Thijs, R. D. , Surges, R. , Brien, T. J. , & Sander, J. W. (2019). Epilepsy in adults. The Lancet, 393(10172), 689–701. 10.1016/S0140-6736(18)32596-0 30686584

[phy215239-bib-0129] Thom, M. (2004). Recent advances in the neuropathology of focal lesions in epilepsy. Expert Review of Neurotherapeutics, 4(6), 973–984. 10.1586/14737175.4.6.973 15853524

[phy215239-bib-0130] Tomkins, O. , Feintuch, A. , Benifla, M. , Cohen, A. , Friedman, A. , & Shelef, I. (2011). Blood‐brain barrier breakdown following traumatic brain injury: A possible role in posttraumatic epilepsy. Cardiovascular Psychiatry and Neurology, 2011, 765923. 21436875 10.1155/2011/765923 PMC3056210

[phy215239-bib-0131] Torres, F. V. , da Silva Filho, M. , Antunes, C. , Kalinine, E. , Antoniolli, E. , Portela, L. V. C. , Souza, D. O. , & Tort, A. B. L. (2010). Electrophysiological effects of guanosine and MK‐801 in a quinolinic acid‐induced seizure model. Experimental Neurology, 221(2), 296–306. 10.1016/j.expneurol.2009.11.013 19948169

[phy215239-bib-0132] Trosclair, K. , Si, M. , Watts, M. , Gautier, N. M. , Voigt, N. , Traylor, J. , & Hamilton, K. A. (2021). Kv1. 1 potassium channel subunit deficiency alters ventricular arrhythmia susceptibility, contractility, and repolarization. Physiological Reports, 9(1), e14702. 33427415 10.14814/phy2.14702 PMC7798052

[phy215239-bib-0133] van Baalen, A. , Vezzani, A. , Häusler, M. , & Kluger, G. (2017). Febrile infection–related epilepsy syndrome: Clinical review and hypotheses of epileptogenesis. Neuropediatrics, 48(01), 005–018. 10.1055/s-0036-1597271 27919115

[phy215239-bib-0134] Ventura‐Mejía, C. , & Medina‐Ceja, L. (2014). Decreased fast ripples in the hippocampus of rats with spontaneous recurrent seizures treated with carbenoxolone and quinine. BioMed Research International, 2014, 1–9. 10.1155/2014/282490. PMC4168142 25276773

[phy215239-bib-0135] Verdugo, C. D. , Myren‐Svelstad, S. , Aydin, E. , Van Hoeymissen, E. , Deneubourg, C. , Vanderhaeghe, S. , & Muto, A. (2019). Glia‐neuron interactions underlie state transitions to generalized seizures. Nature Communications, 10(1), 1–13. 10.1038/s41467-019-11739-z PMC6707163 31444362

[phy215239-bib-0136] Vezzani, A. , Aronica, E. , Mazarati, A. , & Pittman, Q. J. (2013). Epilepsy and brain inflammation. Experimental Neurology, 244, 11–21. 10.1016/j.expneurol.2011.09.033 21985866

[phy215239-bib-0137] Vezzani, A. , Dingledine, R. , & Rossetti, A. O. (2015). Immunity and inflammation in status epilepticus and its sequelae: Possibilities for therapeutic application. Expert Review of Neurotherapeutics, 15(9), 1081–1092. 10.1586/14737175.2015.1079130 26312647 PMC4767891

[phy215239-bib-0138] Vezzani, A. , French, J. , Bartfai, T. , & Baram, T. Z. (2011). The role of inflammation in epilepsy. Nature Reviews Neurology, 7(1), 31–40. 10.1038/nrneurol.2010.178 21135885 PMC3378051

[phy215239-bib-0139] Wallraff, A. , Köhling, R. , Heinemann, U. , Theis, M. , Willecke, K. , & Steinhäuser, C. (2006). The impact of astrocytic gap junctional coupling on potassium buffering in the hippocampus. Journal of Neuroscience, 26, 5438–5447. 16707796 10.1523/JNEUROSCI.0037-06.2006 PMC6675300

[phy215239-bib-0140] Wang, X. , & Li, T. (2020). Role of adenosine kinase inhibitor in adenosine augmentation therapy for epilepsy: A potential novel drug for epilepsy. Current Drug Targets, 21(3), 252–257. 10.2174/1389450119666191014104347 31633474

[phy215239-bib-0141] Wang, Y. , Song, J.‐H. , Denisova, J. V. , Park, W.‐M. , Fontes, J. D. , & Belousov, A. B. (2012). Neuronal gap junction coupling is regulated by glutamate and plays critical role in cell death during neuronal injury. Journal of Neuroscience, 32(2), 713–725. 10.1523/JNEUROSCI.3872-11.2012 22238107 PMC3567463

[phy215239-bib-0142] Wetherington, J. , Serrano, G. , & Dingledine, R. (2008). Astrocytes in the epileptic brain. Neuron, 58(2), 168–178. 10.1016/j.neuron.2008.04.002 18439402 PMC4124883

[phy215239-bib-0143] Wong, M. (2019). The role of glia in epilepsy, intellectual disability, and other neurodevelopmental disorders in tuberous sclerosis complex. Journal of Neurodevelopmental Disorders, 11(1), 1–9. 10.1186/s11689-019-9289-6 31838997 PMC6913020

[phy215239-bib-0144] Wong, M. , & Crino, P. B. (2012). Tuberous sclerosis and epilepsy: Role of astrocytes. Glia, 60(8), 1244–1250. 22438024 10.1002/glia.22326

[phy215239-bib-0145] Xie, N. , Wang, C. , Lian, Y. , Zhang, H. , Wu, C. , & Zhang, Q. (2013). A selective inhibitor of Drp1, mdivi‐1, protects against cell death of hippocampal neurons in pilocarpine‐induced seizures in rats. Neuroscience Letters, 545, 64–68. 10.1016/j.neulet.2013.04.026 23628672

[phy215239-bib-0146] Young, J. D. , Yao, S. Y. , Baldwin, J. M. , Cass, C. E. , & Baldwin, S. A. (2013). The human concentrative and equilibrative nucleoside transporter families, SLC28 and SLC29. Molecular Aspects of Medicine, 34(2–3), 529–547. 10.1016/j.mam.2012.05.007 23506887

[phy215239-bib-0147] Zarrinmayeh, H. , & Territo, P. R. (2020). Purinergic receptors of the central nervous system: Biology, PET ligands, and their applications. Molecular Imaging, 19, 1536012120927609. 10.1177/1536012120927609 32539522 PMC7297484

